# Manipulating the Circadian and Sleep Cycles to Protect Against Metabolic Disease

**DOI:** 10.3389/fendo.2015.00035

**Published:** 2015-03-23

**Authors:** Kazunari Nohara, Seung-Hee Yoo, Zheng (Jake) Chen

**Affiliations:** ^1^Department of Biochemistry and Molecular Biology, The University of Texas Health Science Center at Houston, Houston, TX, USA

**Keywords:** sleep, circadian clock, metabolic disease, obesity, intervention, small molecules

## Abstract

Modernization of human society parallels an epidemic of metabolic disorders including obesity. Apart from excess caloric intake, a 24/7 lifestyle poses another important challenge to our metabolic health. Recent research under both laboratory and epidemiological settings has indicated that abnormal temporal organization of sleep and wakeful activities including food intake is a significant risk factor for metabolic disease. The circadian clock system is our intrinsic biological timer that regulates internal rhythms such as the sleep/wake cycle and also responses to external stimuli including light and food. Initially thought to be mainly involved in the timing of sleep, the clock, and/or clock genes may also play a role in sleep architecture and homeostasis. Importantly, an extensive body of evidence has firmly established a master regulatory role of the clock in energy balance. Together, a close relationship between well-timed circadian/sleep cycles and metabolic health is emerging. Exploiting this functional connection, an important holistic strategy toward curbing the epidemic of metabolic disorders (e.g., obesity) involves corrective measures on the circadian clock and sleep. In addition to behavioral and environmental interventions including meal timing and light control, pharmacological agents targeting sleep and circadian clocks promise convenient and effective applications. Recent studies, for example, have reported small molecules targeting specific clock components and displaying robust beneficial effects on sleep and metabolism. Furthermore, a group of clock-amplitude-enhancing small molecules (CEMs) identified via high-throughput chemical screens are of particular interest for future *in vivo* studies of their metabolic and sleep efficacies. Elucidating the functional relationship between clock, sleep, and metabolism will also have far-reaching implications for various chronic human diseases and aging.

## Introduction

Our bodily functions are temporally coordinated to achieve optimal fitness by an intrinsic biological timer called the circadian clock (1). The clock has evolved in response to the daily rotation of Earth, functioning to drive cycles of metabolism, physiology, and behavior. Among the most fundamental biological cycles is the sleep/wake cycle, in part characterized by profound metabolic changes and alternating energy flux between sleep/fasting and wakefulness/feeding (2, 3). Epidemiological studies of shift workers have provided initial evidence for a greater risk of metabolic disorders correlated with circadian/sleep disruption (4, 5). Furthermore, during natural aging, reduced energy metabolism is accompanied by sleep fragmentation and dampened circadian rhythms in hormone secretion, body temperature, and circadian gene expression (6–8). Such associative evidence strongly suggests a close, reciprocal relationship among the circadian clock, sleep, and metabolism.

**Figure 1:**
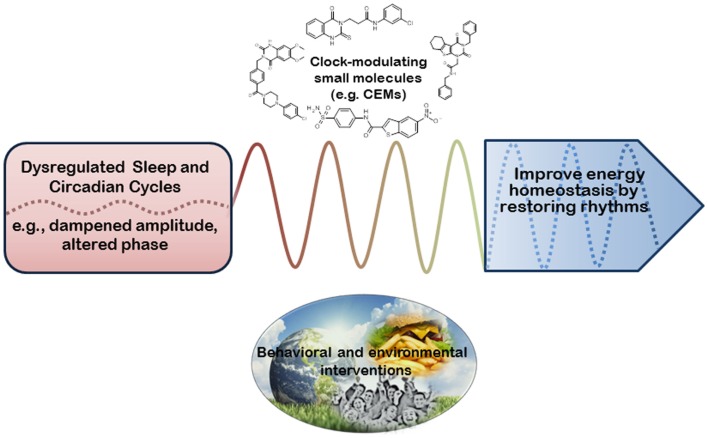
**Biological rhythms-based intervention for metabolic benefits**. Energy metabolism is regulated by both sleep and circadian cycles. Phasic misalignment (e.g., shift work) and/or dampened amplitude of daily behavior (e.g., sleep, food intake) can profoundly impair metabolic homeostasis. Therefore, as opposed to direct manipulation of metabolic regulators, interventional strategies can aim to restore normal phase and amplitude of these biological cycles in order to improve metabolic health. Such interventions may involve behavioral (e.g., meal timing) and environmental (e.g., lighting) means as well as pharmacological agents capable of correcting cycle phase and/or amplitude (e.g., clock-enhancing small molecules, or CEMs). Such rhythm-based interventions may in turn lead to metabolic improvement including favorable energy balance, reduced body weight, and enhanced insulin sensitivity.

Outstanding questions remain concerning the integration of the clock, sleep, and metabolic health. Whereas increasing evidence has demonstrated a complex interplay of molecular mechanisms that the circadian clock employs to regulate metabolic processes (9, 10), the role of sleep in circadian metabolic regulation is less clear (11, 12). Specifically, how sleep disturbances influence energy balance is not well-understood at the molecular level. From the translational perspective, much work is required to fully exploit clock and sleep based interventions against metabolic disorders. In the current review, we will describe temporal control of energy metabolism under both physiological and pathological settings and discuss behavioral, environmental, and pharmacological strategies of targeting circadian/sleep cycles as preventive or therapeutic measures against metabolic disease (Figure 1). We primarily limit our discussion to mammalian species unless otherwise noted.

## Sleep, Circadian Clock, and Metabolism

### Sleep and circadian cycles

Sleep is essential for health and survival (13). Sleep deprivation, depending on duration and severity, can lead to acute impairment of cognitive and physiological functions, increased risks of chronic diseases such as cardiovascular and metabolic diseases, and ultimately fatality (14–17). Despite potentially life-threatening consequences for animals in their natural habitats, sleep clearly serves a vital, evolutionarily conserved function. Mammalian sleep architecture has been characterized in detail by electroencephalograms (EEGs), consisting of rapid eye movement (REM) and non-REM sleep (18). The latter is further divided into stages 1–3, with the deepest slow-wave sleep occurring during stage 3. Sleep is traditionally considered to be regulated by two overlapping processes, the sleep drive (homeostasis, process S) and the circadian timing of sleep (rhythmic sleep propensity, process C) (2, 19). Paradoxically, very little is known about the molecular function of sleep and genetic regulation of sleep homeostasis, which is typically measured by the delta power of slow-wave sleep. Sleep has been proposed to play a role in memory consolidation and metabolic homeostasis involving removal of deleterious byproducts and/or restoration of essential metabolites (3, 18, 20). Interestingly, a recent study demonstrated β-amyloid (Aβ), a neurotoxic peptide accumulated in Alzheimer’s disease, was actively cleared from sleeping brain, highlighting a critical function of the sleep/wake cycle for metabolic detoxification (21).

Timing of the sleep/wake cycle, as well as numerous other physiological processes, is known to be regulated by the circadian clock, our intrinsic biological timer (22–24). The clock is self-sustained, but subjected to resetting by external cues including light and food (25). In mammals, light is transmitted through the retinohypothalamic tract (RHT) to the suprachiasmatic nuclei (SCN), the master pacemaker of the clock system. Through neuronal and hormonal signals such as TGF-α and prokinectin-2, the SCN synchronizes and orchestrates peripheral clocks in individual tissues throughout the body (26). The basic clock unit is the molecular oscillator, ubiquitously present in almost every cell in our body (1). The oscillator is composed of interlocked feedback loops. In the core loop, positive factors (CLOCK/NPAS2, BMAL1) drive the transcription of genes encoding the negative components CRYs and PERs (CRYPTOCHROME1/2, PERIOD1/2), which in turn heterodimerize to inhibit their own transcription. *Bmal1* transcription is further regulated by competing nuclear hormone receptors including retinoid-related orphan receptors (RORs) as positive regulators and reverse-ErbA (REV-ERBs) as negative regulators in the secondary stabilization loop, ultimately generating 24-h molecular oscillation. The molecular oscillator drives expression of the so-called clock-controlled genes (CCGs) in a tissue-dependent manner, which subsequently controls metabolic, physiological, and behavioral outputs (27). Genetic studies in recent years have also provided evidence for important roles of clock genes in sleep homeostasis (22) (see below), indicating a possible interdependence of processes S and C via clock genes.

Melatonin is a pineal gland-derived hormone playing an important role at the interface of the sleep/wake cycle and the circadian clock (28). Melatonin levels display a clear circadian pattern, peaking at night to promote sleep and reaching the trough in the morning and remaining low during the day (29). In accordance, the melatonin biosynthesis pathway, including the key enzyme aralkylamine *N*-acetyltransferase (AANAT), has been shown to be subjected to clock control (30). Furthermore, light exposure also suppresses melatonin synthesis and secretion, mediated by the superior cervical ganglion through the SCN (31). In blind subjects devoid of light perception and exhibiting longer free-running rhythms with an average of 24.5 h, rhythmic melatonin treatment was able to entrain them to approximately 24.0-h periods and concomitantly improve their sleep amount and quality (32). These studies strongly suggest the importance of melatonin for both circadian periodicity and sleep timing, amount, and quality.

### Temporal control of energy metabolism

The necessity to temporally coordinate metabolic events is most apparent in photosynthetic organisms such as cyanobacteria and plants where oxygen-generating photosynthesis must be segregated from oxygen-sensitive nitrogen fixation (33). In fact, redox cycles have recently been shown to operate in diverse phylogenetic branches, presumably evolved following the great oxygenation event (GOE) approximately 2.5 billion years ago (34–36). Particularly, much evidence has recently emerged concerning temporal control of energy metabolism in mammals (37). Epidemiological studies have shown increased risk of weight gain and insulin resistance in shift workers (4, 5, 38). In accordance, human subjects placed under controlled quasi-circadian schedules (28-h days; sleep:wake = 1:2) in a laboratory setting also suffered metabolic deficits characteristic of a prediabetic state within only 10 days of exposure (39), providing a dramatic example of acute influence of misaligned circadian cycles on metabolic well-being. In mice, disruption of circadian cycles, via either genetic or environmental means, has been shown to elicit metabolic disorders (9). For example, *Clock*^Δ^*^19/^*^Δ^*^19^* mutant mice were hyperglycemic and prone to body weight gain either under high-fat diet challenge or later in life (40). Importantly, these mice also displayed disrupted circadian rhythms in eating and activity, concordant with the compromising effects of the mutation on the circadian oscillator (41). Furthermore, *Bmal1*-null mice were also found to be obesity prone when fed with high-fat diet at a young age before confounding phenotypes emerged (42). Consistent with these genetic mutation phenotypes, mice that were artificially subjected to lighting conditions designed to cause misalignment and/or amplitude dampening exhibited body weight gain and other metabolic consequences (43). Together, these studies strongly indicate a close correlation between circadian cycle disruption and metabolic disorders including obesity.

Consistent with the above loss-of-function evidence indicating a temporal regulation of metabolism, profiling studies have supplied high-resolution views of a prevalent circadian oscillation of mRNA, protein, and metabolites in various tissues (27, 44–47). One important finding is that the clock controls metabolic pathways by selectively targeting key steps and components (48). Numerous mechanistic studies since have provided molecular evidence for this mode of “rate-limiting step” regulation. For example, levels of NAD+, an important metabolite for redox balance and a key cofactor for metabolic regulators including SIRT1 and Poly(ADP-ribose) polymerase 1, oscillate in a circadian manner (49). Molecular analysis subsequently showed that the promoter of the gene encoding nicotinamide phosphoribosyltransferase (NAMPT), the rate-limiting enzyme catalyzing the formation of nicotinamide mononucleotide from nicotinamide and 5′-phosphoribosyl-pyrophosphate during NAD+ biosynthesis, contains a canonical E-box recognized by CLOCK/BMAL1 and thus tightly controlled by the circadian clock (50–52). Interestingly, NAD+ rhythms have been shown to control mitochondrial oxidative metabolism such as fatty acid oxidation, in part through deacetylation of oxidative enzymes by SIRT3 (52).

The circadian metabolic regulation also involves systemic control by the central nervous system including the hypothalamus and the brainstem (25, 53). In the hypothalamus, multiple cell bodies have been shown to play an important role in endocrine regulation and energy homeostasis, including arcuate nucleus (ARC), paraventricular nucleus (PVN), lateral hypothalamic area (LHA), and dorsomedial hypothalamus (DMH) (54, 55). Importantly, the SCN master pacemaker interacts with these extra-SCN energy centers to exert central control over circadian metabolism. For example, SCN neurons form reciprocal connections with ARC neurons expressing orexigenic neuropeptide Y/Agouti-related protein (NPY/AgRP) and anorexignenic pro-opiomelanocortin/cocaine and amphetamine-regulated transcript (POMC/CART) peptides (56, 57). Furthermore, SCN neurons and nutrient-sensing neurons in the ARC and ventromedial hypothalamus (VMH) project to DMH either directly or indirectly, and employ the melanocortin system to regulate thermogenesis, sleep, corticosteroid secretion, wakefulness, and feeding (53, 55). In addition, SCN neurons also innervate the LHA where orexin (ORX, also known as hypocretin) neurons are located (53). ORX is a neuropeptide hormone which stimulates arousal and energy expenditure (37, 58). In particular, the ORX signaling pathway serves a pivotal function in the balancing action of the ventrolateral preoptic nucleus (VLPO)/extended VLPO (eVLPO) of the hypothalamus and the tuberomammillary nucleus (TMN)/raphe/locus coeruleus (LC) in the hypothalamus and the brainstem (55). ORX-deficient mice suffered from narcolepsy, and interestingly also became obese due to reduced energy expenditure despite a concomitant impairment in food intake (59).

### Sleep restriction as a risk factor for obesity and energy imbalance

A growing body of evidence supports a functional correlation between sleep restriction (shorter duration and/or poor quality) and metabolic disorders, particularly obesity and insulin resistance (4, 38, 60, 61). As mentioned above, epidemiological studies have unveiled an increased prevalence of metabolic syndrome in night/shift workers who stay up at night and sleep during the day (4). For example, among female nurses studied, rotating shift-work duration positively correlated with a trend of increase in body mass index (BMI) (62) and development of type 2 diabetes (63). Under laboratory conditions, partial or total sleep deprivation over a short time period was found to increase energy expenditure (64, 65); however, insufficient sleep provoked hyperphagia to override the enhanced energy need and thus increase the risk of exaggerated body weight gain and obesity (65).

An important physiological substrate mediating sleep regulation of energy balance is the neuroendocrine system (55). For example, during slow-wave sleep, brain glucose metabolism is diminished, which tightly correlates with a reduction in the whole body glucose metabolism (66). This in turn corresponds to attenuated sympathetic nervous activity and reciprocally augmented secretion of growth hormones. Conversely, deprivation of nocturnal sleep in normal humans led to lower post-sleep/-bed time increase of insulin secretion rate (ISR) and also reduction in growth hormone secretion relative to subjects with normal sleep (67). A similar decrease of ISR was also observed during daytime recovery sleep compared with nocturnal sleep (67), suggesting poor sleep quality associated with abnormal timing (diurnal sleep) can negatively impact hormonal regulation of energy homeostasis (68). Furthermore, sleep time restriction has also been shown to have deleterious effects on glucose homeostasis (64, 67). For example, sleep limitation at 4-h time-in-bed (TIB) for six nights was found to compromise glucose tolerance (12, 61). Interestingly, even two nights of 4-h TIB was sufficient to cause significantly elevated ghrelin:leptin ratio (by approximately 70%), indicating a strong propensity for greater food intake (69). Despite certain experimental incongruence (64), a general role of sleep in neuroendocrine regulation of energy metabolism is well accepted.

Many important questions remain to be further investigated. For example, we still lack mechanistic understanding at the molecular or cellular level. In particular, little is known with regard to the relationship between sleep restriction and circadian disruption in the context of metabolic disease. Specific manipulation in the circadian clockwork can lead to defined circadian phenotypes, and consequently a clear causal relationship between circadian cycle and sleep (see below). However, due to limited molecular understanding of the sleep circuit, sleep restriction by behavioral and environmental means is invariably accompanied with altered circadian rhythms, precluding an unequivocal delineation of functional causality. Furthermore, the functional consequence specific for sleep shortening or impairment is not always clear, especially in epidemiological studies (12).

### Core clock genes in the regulation of sleep and metabolism

Molecular, genetic, and physiological characterization of core clock genes have revealed profound influence of circadian timing on both sleep and metabolism (22, 23, 53). An elegant example of circadian gene function in sleep disorder was illustrated by studies of familial advanced sleep phase disorder (FASPD, previously called FASP syndrome) (23). Affected individuals display Mendelian circadian rhythm and sleep phenotypes characterized by excessive phase advances in sleep/wake cycles (e.g., going to bed at 7:30 p.m.). Two mutations have been identified as genetic basis for FASPD, including a serine-to-glycine mutation in human PER2 (70, 71) and a threonine to alanine mutation in casein kinase Iδ (CKIδ), a kinase known to phosphorylate PER2 prior to ubiquitin-mediated PER2 degradation (72–74). Interestingly, the *Per2* mutation site serine, S662, was the first serine in a five-serine phospho-cluster, and appeared not to be a substrate site for CKI kinases. More recent work on *Drosophila* PER protein, to which PER2 is most homologous, identified NEMO as the priming kinase for dPER, functioning to promote subsequent phosphorylation events by the *Drosophila* casein kinase double-time (DBT) and ultimately proteasomal degradation (75). Together, these genetic and molecular studies highlight a key role of *Per2* in the regulation of sleep phase. Interestingly, the mammalian *Per3* gene, homologous to *Per2* (76), has also been shown to play a role in sleep phase and homeostasis control in mouse knockout and human polymorphism studies (77, 78).

More recently, familial natural short sleepers (FNSS) were found to harbor a mutation in the gene encoding the circadian transcriptional repressor DEC2 (79). DEC2 and its homolog DEC1 were initially found to regulate *Per1* gene transcription (80), and mouse studies have provided evidence for their role in circadian phase, resetting in response to light pulses (80–82). The P385R DEC2 mutation identified in FNSS was shown to diminish its transcriptional repression in reporter assays. Importantly, whereas FASPD patients do not exhibit deficits in sleep homeostasis (83), FNSS suffers clear sleep deprivation (79), suggesting a role of circadian genes in sleep homeostasis. This study thus adds to a growing body of genetic evidence implicating various clock genes in the regulation of the homeostatic process of sleep (22, 84, 85).

Pioneering studies showed that SCN lesion led to sleep fragmentation, consistent with a role of the clock in sleep timing and architecture (86). However, the homeostatic recovery subsequent to sleep deprivation appeared not to be affected, suggesting that clock gene expression in the SCN may not affect sleep homeostasis. Considering the close relationship of both sleep and peripheral (non-SCN) clocks with metabolic well-being, it has been postulated that metabolism may form the physiological basis for peripheral clock regulation of sleep homeostasis (22, 53). In other words, whereas the circadian clock can directly govern sleep timing (Process C), the homeostatic control (Process S) may be regulated by the clock indirectly via circadian metabolic pathways (10). Alternatively, it is also possible that different clock genes may in fact serve distinct non-clock functions to regulate sleep. This notion is supported by divergent phenotypes in circadian mouse models that are not easily compatible in the strict context of the clock. One notable case is the severe phenotype observed in *Bmal1* knockout mice (87). In addition to a loss of circadian rhythmicity, the mice displayed profound developmental, metabolic, and pre-mature aging phenotypes not characteristic of other arrhythmic mouse models (76, 88–90).

**Figure 2:**
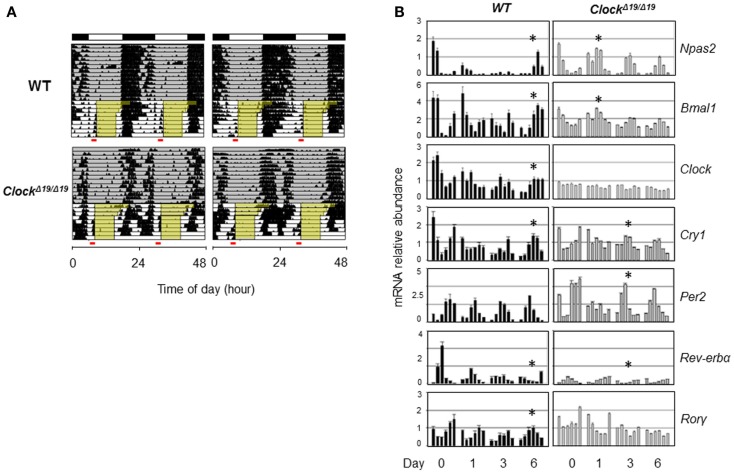
**Behavioral and molecular responses of *Clock*^Δ^*^19/^*^Δ^*^19^* to restricted feeding**. **(A)** Double-plotted actograms of C57B/6 wild-type (WT) and *Clock*^Δ^*^19/^*^Δ^*^19^* mice under 12L:12D conditions as denoted in the bars at the top. Ad lib food availability is indicated by the gray shading. Step-wise food restriction was carried out from 12 to 8 h (indicated by yellow shading), and the food anticipatory activities (FAA) were detected just prior to food availability (marked by red bars). Two representative actograms are shown for each genotype. **(B)** Mice were sacrificed on the indicated days of restricted feeding as in **(A)**. Liver samples were collected from three mice per genotype on each of the indicated six time points, every 4 h across the circadian cycle starting from ZT0 (light on). Total RNAs were extracted from these samples and expression of circadian core clock genes are quantified by real-time qPCR analysis. Asterisks indicate the time when circadian phase was reversed by restricted feeding.

Genetic background and the nature of the genetic mutation should also be taken into account when considering the role of clock genes in sleep. CLOCK and NPAS2 are paralogous basic helix–loop–helix PAS domain-containing transcription factors (88, 91). The classical *Clock*^Δ^*^19/^*^Δ^*^19^* mutant mice, expressing a dominant negative version of CLOCK deficient in transactivation yet still able to interact with its heterodimeric partner BMAL1, displayed severely compromised circadian rhythms and suffered from hyperphagia and defective glucose and lipid homeostasis (40). Interestingly, these mice also exhibited altered sleep homeostasis under both baseline conditions and sleep recovery following deprivation; in particular, REM sleep rebound over 24 h following sleep deprivation was reduced by half in *Clock*^Δ^*^19/^*^Δ^*^19^* mice (92). When compared with wild-type (WT) mice, *Clock*^Δ^*^19/^*^Δ^*^19^* mice showed normal or even accelerated food anticipatory activities (FAA) in response to restricted feeding (93) (Figure 2A), and time-course analysis of core clock gene expression under the restricted feeding condition revealed concordantly accelerated phase shift (Figure 2B). In comparison, *Npas2*-deficient mice were largely normal in circadian behavioral rhythmicity (90). However, these mice showed a unique phenotype wherein the siesta sleep in the second half of the active period was largely absent as indicated by actogram recording (90). Detailed analysis further revealed a role of *Npas2* in maintaining normal NREM sleep time (94). In accordance with an important metabolic sensing function of NPAS2 (10), *Npas2*-deficient mice were ill-adaptive to restricted feeding, as indicated by a delayed FAA response (90). It remains unclear whether CLOCK possesses a metabolic sensing function similar to that of NPAS2 or whether the distinct FAA responses in *Clock*^Δ^*^19/^*^Δ^*^19^* and *Npas2*-deficient mice were due to differences in circadian rhythmicity or other gene-specific effects such as target gene expression (95). Overall, future research is needed to delineate the roles of specific clock genes in sleep regulation, particularly in the context of metabolic function of the clock.

## Modulating Sleep and Circadian Cycles for Metabolic Benefits

### Behavioral and environmental interventions

Dieting and physical exercise are common interventions to restore energy balance favoring expenditure over intake, thereby guarding against obesity and other metabolic disorders (96). Importantly, meal timing is increasingly appreciated as an important temporal parameter for hormonal balance and energy expenditure. In shift workers, sustained disruption of the intrinsic/environmental synchrony leads to abnormally timed meals. Although the total daily caloric intake remained constant, glucose and lipid homeostasis was disrupted, correlated with increased risks of obesity in shift workers (97). Furthermore, in night-eating syndrome (NES), late night eating, and consequently significant nighttime calorie intake leads to disruption of the sleep/wake cycle; importantly, patients are also prone to developing obesity (98). Laboratory rodent studies have also provided experimental evidence in support of a key role of meal timing and metabolic health. For example, daytime-limited feeding in nocturnal mice resulted in greater body weight gain compared with normal nighttime feeding (99).

More recent mouse studies further underscore an interventional role of meal timing. In one study, time-restricted feeding (TRF) of high-fat diet for 8 h daily during the dark phase was compared with *ad lib* conditions (100). Although the calorie intake was comparable, the TRF regimen conferred a striking protective function against metabolic disorders including obesity and hyperinsulinemia. Importantly, TRF was found to enhance nutrient mobilization and energy expenditure, consistent with the notion that the obesity risk associated with abnormal meal timing may result from the inability for efficient and timely energy expenditure. The authors further explored the metabolic efficacy of TRF using mice fed with different obesogenic diets, including high-fat, high-fructose, and high-fat/high-sucrose diets (101). Remarkably, in both preventive and therapeutic experimental designs, TRF demonstrated robust efficacy in reducing body weight gain and generally protecting mice from potential or existing metabolic disorders. Several important questions remain to be answered, including the underlying mechanisms likely involving diet-specific circadian pathways, the efficacy in humans and importantly the effects on sleep and other behavioral rhythms. Regardless, these studies highlight a simple behavioral intervention potentially applicable to a diverse array of metabolic challenges.

For millennia, human daily life was dictated by the “natural” light and dark cycles. However, technological and social modernization over the past century drastically altered our lifestyle, and one of the most profound consequences is constant exposure to artificial bright light. On the one hand, bright light therapy is widely used to guard against mood disorders (102). On the other, light can entrain not only the central circadian pacemaker but also peripheral clocks via the sympathetic nervous system (103). Therefore, light exposure in the evening can result in misalignment between the sleep/wake cycle and the intrinsic circadian rhythms in night/shift workers (104), which may in turn increase the incidence rate of the metabolic syndrome. Interestingly, even dim light exposure at night for nocturnal rodents was found to carry significant metabolic consequences (105). Specifically, male mice exposed to dim light at night (LAN) displayed altered meal timing over the circadian cycle, consuming significantly elevated calories during the daytime. These mice consequently showed greater body weight gain, an effect reversible by restricted feeding during the active phase under the LAN conditions (43, 106). In addition to circadian timing, light wavelength is also important; for example, the circadian rhythm of melatonin is more sensitive to photic resetting at a short wavelength of 460 nm compared with 555 nm (107). These studies together suggest that an optimal lighting condition (source and timing) may play an important role in behavioral circadian rhythms and concomitantly prevent metabolic disease.

### Metabolic effects of common hypnotics

Although behavioral interventions are usually relatively inexpensive and easy to implement, the outcome requires high patient compliance. Specifically, for metabolic disease, lifestyle changes focusing on improving dietary quality, physical activity, and sleep habits can serve as a first-line treatment but are often unsuccessful clinically due to poor compliance (108). Therefore, it is important to develop pharmacological agents, which normally do not require drastic and prolonged lifestyle modification and thus promise greater patient motivation (109).

Table 1 lists several major classes of sleep medicine and their reported metabolic effects. Besides playing an important role for circadian/sleep cycles, melatonin is also a key metabolic regulator, previously shown to regulate pancreatic insulin secretion and glucose transport (110). In accordance, human studies have provided association evidence supporting a direct relationship between melatonin levels and the risk of developing type 2 diabetes (111), suggesting a causal relationship between reduced melatonin levels, either by sleep debt or shift-work, and metabolic disease. As a result, recent studies using animal models and humans have begun to explore the therapeutic efficacy of melatonin for the metabolic syndrome (112, 113). A number of small-molecule agonists for melatonin receptors have also been identified (114, 115). Interestingly, consistent with the reported suppressive effects of melatonin on body weight gain, newly identified melatonin agonists were also found to improve energy metabolism and insulin sensitivity (114–116). For example, in obese rats fed with high-fat/high-sucrose diets for 5 months, treatment of NEU-P11 via intra-peritoneal injection was able to blunt weight gain, reduce abdominal fat, and improve glucose homeostasis (114, 115) (Table 1). This metabolic efficacy is concordant with a genome-wide association study (GWAS) of European subjects where a single nucleotide polymorphism (SNP) rs1387153, localized near the melatonin receptor 2 (MTNR1B) locus, was identified as a key modulator of fasting blood glucose (117), together suggesting an important role of melatonin signaling in mediating the metabolic regulation by the sleep/circadian clock. In comparison, olanzapine, a promiscuous ligand for both gamma-aminobutyric acid receptor type A (GABA_A_) and histamine H_1_ receptors, was found to adversely affect energy metabolism, including body weight gain and altered plasma glucose and lipid levels (118) (Table 1). Therefore, metabolic efficacy, beneficial, or adverse, of sleep medicines should be characterized for individual drugs.

**Table 1:** **Metabolic effects of commonly used hypnotic drugs**.

Hypnotics	Metabolic effects
**GABA_A_ receptor agonists**	**Olanzapine** (2-methyl-4-(4-methyl-1-piperazinyl)-10H-thieno [2, 3-b][1, 5]benzodiazepine)
*Benzodiazepine*	Overweight, hyperglycemia, hyperinsulinemia, dyslipidemia, ketoacidosis, and visceral fat accumulation (119, 120)
*Non-benzodiazepine barbiturate*	Increased plasma TG, HDL-cholesterol levels, higher cholesterol/HDL-cholesterol ratios, hyperinsulinaemia (118)
	**Zolpidem** (*N*,*N*-dimethyl-2-(6-methyl-2-*p*-tolylimidazo[1,2-a]pyridin-3-yl)acetamide)
	Increased body weight, decreased locomotor activity, and food intake (121)
**H_1_ receptor antagonists**	**Prescription H_1_ antihistamines**
	Increased body weight, BMI, waist circumference and serum insulin levels (122), exacerbated high-fat diet-induced hepetic steatosis (123)
Antihistamines	**Olanzapine**
	Increased body weight, food intake, fat mass, and fat cell number (124)
**Melatonin and melatonin receptor agonists**	**Melatonin** (N-acetyl-5-methoxytryptamine)
	Reduced fat mass, body weight, and improve insulin sensitivity (125, 126)
	Improved BMI, blood pressure, and lipid profile (127)
	Resistance to diet-induced obesity with time dependent (116)
	**Rozerem** (*S*-*N*-[2-(1,6,7,8-tetrahydro-2*H*-indeno-[5,4-*b*] furan-8yl) ethyl]propionamide)
	Improved age-associated hypertension and weight gain (128)
	**Piromelatine** (Neu-P11; N-(2-(5-methoxy-1H-indol-3-yl)ethyl)-4-oxo-4H-pyran-2-carboxamide)
	Reduced body weight gain, improved insulin sensitivity under DIO (114)
	Improved insulin sensitivity under chronic sleep restriction (115)
**Serotonin (5HT) receptor antagonists**	***m-chlorophenylpiperazine (mCPP)*** (1-(3-chlorophenyl)piperazine hydrochloride)
	Improved glucose tolerance and insulin sensitivity (129)
	**Lorcaserin** (1*R*-8-chloro-1-methyl-2,3,4,5-tetrahydro-1*H*-3-benzazepine)
	Reduced of body weight, improve blood lipid profile, improved blood pressure (130)
**Orexin receptor antagonists**	**SB-334867** (1-(2-methylbenzoxazol-6-yl)-3-[1,5]napthydrin-4-yl urea hydrochloride)
	Reduced food intake, fat mass, body weight, and increased energy expenditure (131)
	**ACT 335827** (αR,1S-1-[(3,4-Dimethoxyphenyl)methyl]-3,4-dihydro-6,7-dimethoxy-N-(1-methylethyl)-α-phenyl-2(1H)-isoquinolineacetamide)
	Increase water intake and HDL in DIO mice (132)
	**SB-408124** (1-(6,8-difluoro-2-methyl-quinolin-4-γl)-3-(4-dimethylamino-phenyl)-urea)
	Reduced body weight (133)

A number of endogenous metabolites, including adenosine, have been shown to induce sleep in rodents (18, 20, 134). Moreover, high-throughput screening using behavioral assays has also led to identification of novel small-molecule modulators of sleep. In a fly based sleep screen of 1280 bioactive compounds (135), reserpine, an inhibitor of the vesicular monoamine transporter (VMAT) functioning in the formation of monoamine-containing presynaptic vesicles, displayed a sleep-inducing activity based on locomoter behavior assays. In accordance, VMAT mutant flies showed enhanced sleep and arousal threshold levels. Likewise, a similar rest/wake locomotor behavioral assay in larval zebrafish was adopted to screen 5648 structurally divergent compounds (136). Strikingly, approximately 10% of the compounds were found to impact certain sleep parameters to varying degrees. In addition to conserved sleep-regulatory targets such as adrenergic receptors, this study also identified novel molecular targets including Ether-a-go-go Related Gene (ERG) potassium channels. Future studies will be needed to address metabolic functions and adverse effects of the newly identified sleep modulating compounds. Given the divergence of sleep between mammalian and non-mammalian species, it remains to be seen whether the identified novel targets and/or small-molecule modulators can be extrapolated to mammals. Ultimately, a direct mammalian sleep screen, albeit challenging, will likely yield unique insight.

### Circadian clock-modulating small molecules

With greater appreciation of a broad and fundamental role of the circadian clock in various pathophysiologies, several recent studies have reported identification of clock-modulating small molecules (1, 137–139). Compared with the traditional chronotherapy where dosing of drugs is purposely aligned to a certain circadian time window to maximize the therapeutic index, such small molecules allow direct molecular manipulation of the circadian clock for beneficial effects in physiology and behavior.

Clock-modulating small molecules can be developed either by targeting a defined clock component of interest or via circadian phenotypic screening (138, 139). Several circadian components have been exploited for specific ligand development, most notably CKI and the antagonizing nuclear hormone receptors REV-ERBs and RORs. A number of CKI inhibitors have previously been characterized, displaying cross-inhibition of multiple CKI isoforms (e.g., CKIδ and CKIε) (140, 141). Surprisingly, an isoform-specific inhibitor of CKIε, PF-4800567, was found to exert minimal effects on circadian periodicity (142, 143), in support of a major role of CKIδ in clock regulation. These studies serve as proof of principle to further exploit the CKIε-specific ligand in clock-related physiology, particularly in light of a demonstrated CKIε function in sleep (144).

A number of ligands for REV-ERBs and RORs have been reported in recent years (139, 145). A series of studies focused on SR9011, a REV-ERB agonist developed through targeted chemical modification (146). SR9011 was found to alter clock gene expression in metabolically active tissues and transiently repress circadian wheel-running behavior. SR9011 treatment in diet-induced obese mice led to improved metabolic parameters including body weight and serum lipid and glucose levels (146). More recent work further demonstrated that SR9011 regulates sleep, increasing wakefulness in treated mice and reciprocally suppressing REM and slow-wave sleep (147). The latter study also reported an anxiolytic effect of SR9011 in WT but not *Rev-erb*β knockout mice. These findings together highlight a promising potential of REV-ERB agonists in modulating circadian/sleep-related metabolism and behavior. Small molecules targeting RORs and other clock components such as the melanopsin receptor have also been described (139, 148).

Small-molecule identification via phenotypic screens entails high-throughput based circadian reporter assays, most commonly driven by *Per2* and *Bmal1* promoters (138, 141, 149). Given the robust precision in circadian periodicity measurement, a great majority of clock-modulating small molecules were identified based on their effects on circadian period length (138). Highlighting a predominant regulatory role of CKI in circadian periodicity, the most pronounced period-lengthening effects are primarily associated with CKI inhibitory compounds (140, 141, 150). These studies complement the molecular genetic finding that PER protein half-life is a key determinant of the period length (74). Likewise, CRY proteins, dimerizing with PERs in the negative arm of the core loop, are also subjected to elaborate protein stability control, and mutations in two paralogous E3 ligases for CRYs have been discovered in mouse forward genetic screens to cause period changes (151). KL001, a period-lengthening compound identified by unbiased phenotypic screen, was recently shown to directly interact with CRY proteins and consequently interfere with their degradation (152).

Recent studies have also revealed a group of clock-amplitude-enhancing small molecules (CEMs) dubbed (138, 150). For example, four CEMs were previously found to enhance cellular and tissue reporter rhythms in both WT and *Clock*^Δ^*^19/^*^+^ heterozygous mutant background (150). These molecules were not able to reinvigorate fully, or at least severely, disrupted rhythms in *Clock*^Δ^*^19/^*^Δ^*^19^* homozygous or *Bmal1*-null cells (138). For practical purposes, however, this is not a major limitation as null clock phenotypes are rare in human populations. Interestingly, one of the CEMs exhibits a unique quality of enhancing SCN rhythms (150), known to be highly recalcitrant to genetic and environmental perturbations due to robust neuronal coupling (153, 154). While acute jet-lag can be best remedied when the clock is disrupted or with low amplitude (155), for chronic diseases and aging known to display dampened circadian rhythms (7, 8, 156–158), it is tantalizing to speculate that CEMs can help retard or even reverse physiological decline and improve metabolic and physiological well-being in animals and humans. For example, sleep fragmentation is a hallmark of aging and also frequently observed in age-related diseases including Alzheimer’s disease (159). It is characterized by multiple short periods of sleep during normal sleep timing and sleep during usual active phase, thus exemplifying dampened amplitude of the sleep/wake cycle. It is of great interest to investigate a putative role of CEMs in sleep and also metabolism. At the mechanistic level, the molecular basis for clock amplitude, or robustness, is not as well-understood compared with circadian period and phase regulation (160). Besides the well-characterized nuclear hormone receptors in the stabilization loop of the core oscillator (REV-ERBs and RORs) (25), genomic siRNA screen studies have revealed hundreds of genes that, when knocked down, enhanced or repressed the oscillatory amplitude of reporter rhythms, indicating a broad systemic control of clock amplitude (161).

## Concluding Remarks

Exciting advances in the past decade or so have revealed much molecular insight into the circadian regulatory mechanism of energy metabolism (10, 37). Existing evidence also supports a causal role of sleep disruption or restriction in the development of the metabolic syndrome including obesity (12), although the mechanistic basis is not well-understood. As the research community continues to decipher genetic and molecular function and mechanism of sleep, behavioral, environmental, and pharmacological intervention strategies are being actively pursued to manipulate circadian and sleep rhythms to optimize cellular energetics (Figure 1). This novel approach exploits the translational potential of biological rhythms to combat the continuing rise of the global metabolic disease epidemic in our modern society, complementing the more “direct” strategy aiming to correct particular metabolic regulators or pathways.

The metabolic regulation by biological rhythms is important beyond the realm of the metabolic disease. Other pathologies are also characterized by strong temporal components and intimately associated with dysregulated metabolism (1). For example, the morning surge of blood pressure is a well-documented culprit for cardiovascular disease related sudden death (162, 163). The circadian/sleep cycles also play an important role in cardiometabolic function, as illustrated by increased hypertensive risks in circadian clock mutant mice, forced desynchrony in laboratory studies, as well as shift workers (163–165). No disease has a greater penetrance than aging – we all age. In addition to the aforementioned sleep and circadian deficits, age-associated decline also encompasses deterioration of energy homeostasis, physical performance and cognitive function, which may eventually manifest in chronic and degenerative diseases (6–8, 156, 166). Application of biological rhythm-based intervention strategies to these disease targets is of great potential and will likely lead to exciting advances in the near future.

## Conflict of Interest Statement

The authors declare that the research was conducted in the absence of any commercial or financial relationships that could be construed as a potential conflict of interest.
